# Diversity and Composition of Gut Microbiota in Different Developmental Stages of the Tibetan Toad (*Bufo tibetanus*)

**DOI:** 10.3390/ani15121742

**Published:** 2025-06-12

**Authors:** Kaiqin He, Cong Han, Chenyang Liu, Lixia Zhang

**Affiliations:** 1 Department of Ecology, College of Life Sciences, Henan Normal University, Xinxiang 453007, China; htu_hkq@163.com (K.H.); htu_hc@163.com (C.H.); htu_lcy@163.com (C.L.); 2 Puyang Field Scientific Observation and Research Station for Yellow River Wetland Ecosystem, Puyang 457183, China

**Keywords:** *Bufo tibetanus*, Illumina MiSeq sequencing, intestinal microbiota, development stages

## Abstract

The intestinal microbiota is vital for host immunity and metabolism, and its changes are associated with the development stage of hosts. However, little is known regarding how growth and development of anurans affect the diversity of their microbiota, which has a complex life cycle. The Tibetan toad (*Bufo tibetanus*) is a wild population in the high-altitude area of southwest China, which has special adaptability to the environment. Here, the microbial community of the Tibetan toad at six developmental stages (from the tadpole at Gosner stage 18 to the 8-year-old adult) was assessed using high-throughput 16S rRNA sequencing. The alpha diversity index analysis showed that the Chao, Ace, and Shannon indices were highest at Gosner stage 32 and decreased as development progressed, and their alpha diversity remained unchanged over time in adult stages. Beta diversity revealed that the gut microbiota structure differed significantly from Gosner stages 18 to 31, and it became similar to adult toads from Gosner stages 45 to 46 and in juvenile groups. At the phylum level, Firmicutes, Proteobacteria, and Actinobacteria were dominant phyla in tadpoles and adults. The relative abundance of Firmicutes and Proteobacteria in the adult group was significantly higher and lower than that of tadpoles, respectively. The linear discriminant analysis effect size (LEfSe) analysis identified seven phyla exhibiting significant differences during life stages: Verrucomicrobiota, Bacteroidota, and Proteobacteria (Gosner 18 to 31), Cyanobateria and Chloroflexi (Gosner 32 to 41), Actinobacteriota (Gosner 45 to 46), Desulfobacterota (juvenile group), and Firmicutes (adult group). A pathway enrichment analysis revealed that the metabolism and biosynthesis of secondary metabolites were significantly enriched across all developmental stages. This research unveiled variations in the intestinal microbiota composition during development in anurans. Factors such as developmental stage, habitat type and feeding habit jointly affected the gut microbial diversity and community composition in the Tibetan toad. The findings of this study can provide information for understanding the influence of historical developments on the intestinal microbiota and provide protection information for anurans.

## Simple Summary

We studied the intestinal microbiota of *Bufo tibetanus* at different growth and development stages. Through 16S rRNA sequencing and bioinformatics analysis, significant differences were found in gut microbiota diversity (alpha and beta diversity) at different growth stages. The relative abundance of Firmicutes increased continuously with the growth of toads, reaching its maximum in the adult group, and Desulfobacterota was the most abundant phylum in the juvenile group. *Bacillus* and *Succinispira* had the highest relative abundance values at Gosner 18 to 31 stages. An LEfSe analysis identified specific gut microbiota at different stages, such as Proteobacteria at Gosner 18 to 31 stages, Desulfobacterota in the juvenile group, and Firmicutes in the adult group. A functional prediction analysis illustrated that the metabolic pathways and the biosynthesis of secondary metabolites were significant enrichment in all stages. These results indicated that the intestinal microbiota of *B. tibetanus* had adaptive changes at different growth stages, which provided insights for future research on the intestinal microbiota of amphibians.

## 1. Introduction

Gut microbiota is one of the main components of the intestinal ecosystem and can profoundly influence the biology of hosts, including digestive efficiency, metabolic processes, and immune regulatory processes [1,2]. In addition, a number of studies have established it contributes to the host’s ability to better adapt to changes in the environment, by aiding in the absorption of essential nutrients and maintaining organism balance [3,4,5,6]. It is well recognized that developmental changes in gut microbiota composition are mainly driven by host factors [7,8,9,10,11], such as the host genotype, gender, and diet [12,13,14,15,16,17,18]. Furthermore, the developmental stage of the host can also influence gut microbiota in animals, which has been confirmed in mammals [19,20,21,22], fish [23,24,25], shrimp [26], and artificially raised amphibians [27].

Anurans, noted for their unique life histories, are critical in ecosystems and exhibit enormous variations in life histories [28,29,30]. Many frogs and toads undergo metamorphosis, during which the external and internal body morphologies of tadpoles transform dramatically as they transition to a new ecosystem [31,32,33,34]. Meanwhile, the prey diet of tadpoles shifts from microalgae to insects [35]. In addition, the anurans can survive long winter by reducing the metabolic rate and reshaping the gut microbiota in cold regions, without food intake [36]. Thus, the unique life history of anurans leads to a unique microbial ecology in which gut microbiota can be reshaped by changes in morphological, physiological, environmental, and behavioral factors [27,37], making them an ideal model for examining differences in gut microbiota caused by different growth stages [27].

The intestinal microbial composition of anurans during the tadpole and adult stage is significantly different [37,38,39]. A study on *Lithobates pipiens*’s intestinal microbiota showed that the structure of the tadpole was similar to that of teleost fish, but the adult stage was similar to that of amniotic animals [32]. Moreover, the intestinal microbial richness and diversity in the adult stage were lower than those in the tadpole periods [32]. Further, studies on tadpoles in different regions had similar results [40]. The effects of food, habitat type, and specific life stages (such as metamorphosis and hibernation) on the gut microbial composition of some tadpoles and frogs have been reported [37]. In addition, there have also been many studies on the skin microbiota of amphibians [41,42,43,44,45]. However, systematic studies on the succession of microbiota are limited in anurans [2,38,46,47,48,49,50].

The Tibetan toad (*Bufo tibetanus*) belongs to the Bufonidae family and is endemic to southwestern China [51,52]. It is often found in grassland patches and farmlands, with an elevation range from 2400 to 4300 m asl [53]. The spawning period of this species extends from April to July, and the individuals spawn once in a year [53]. Thus, the toad can be classified as a prolonged breeder. Scientific research on the biology and ecology of *B. tibetanus* has been conducted on its skin structures, male call characteristics, populational age structure, and mitochondrial DNA [54,55,56,57,58]. *B. tibetanus* is a wild population in the high-altitude area of southwest China, which has special adaptability to the environment. Until now, little research on the succession of microbiota has been conducted in this toad. Here, we did so by examining the composition and structure of gut bacterial assemblages in the Tibetan toad across its life cycle. This study will help to expand the understanding of the effects of historical developments on the diversity and structure of intestinal microbial communities in wild anurans.

## 2. Material and Methods

### 2.1. Sample Collection

All the samples were collected from Bomi town in Southeastern Tibet, China (29°53′ N, 95°41′ E, 2650 m asl), which is characterized by a humid subtropical climate. The tadpole, juvenile, and adult stage individuals were collected in July 2022. The developmental stage of each tadpole collected was determined using Gosner’s (1960) table [59]. The tadpoles were divided into 4 groups: T1 group (at 18–31 Gosner stages, *n* = 15), T2 group (at 32–41 Gosner stages, *n* = 15), T3 group (at 42–44 Gosner stages, *n* = 10), T4 group (at 45–46 Gosner stages, *n* = 11). The juveniles and adults were named J group (*n* = 6) and A group (*n* = 25), respectively.

All samples were transported to local laboratory facilities and euthanized by soaking in buffered MS-222 (10 g/L). Then, each animal was dissected, and the contents of the guts were collected and immediately frozen in liquid nitrogen. In order to avoid contamination risks, all dissection instruments were wiped with 100% EtOH and flame-sterilized between each individual sample.

### 2.2. DNA Extraction and Sequencing

DNA was extracted from gut content samples using the MagAtrract PowerSoil Pro DNA Kit (Qiagen, Hilden, Germany) according to the manufacturer’s instructions, and its quality was analyzed by agarose gel. The V3–V4 region of the 16S rRNA gene was amplified using the primer sequences 338F (5′-ACTCCTACGGGAGGCAGCAG-3′) and 806R (5′-GGACTACHVGGGTWTCTAAT-3′) for each gut sample. The amplicon products were gel-electrophoresed, purified using the AxyPrep DNA gel extraction kit (Axygen Biosciences, Union City, CA, USA), and quantified using QuantiFluor™-ST (Promega, Madison, WI, USA). Then, the purified amplicons were pooled at equal nanomolar concentration and sequenced on the Illumina MiSeq Sequencing Platform with paired-end read (2 × 300 bp) (Illumina, San Diego, CA, USA). Using standard protocols, sequencing was performed by Majorbio Company (Majorbio, Shanghai, China).

Raw sequencing data were quality-filtered by fastq (version 0.19.6) and merged by FLASH (version 1.2.7) based on overlapping relationships to yield effective sequences. Representative sequences for each operational taxonomic unit (OTU) were obtained by QIIME1.9.1 software and were compared and annotated with the Silva (Release 138) database. Archaea, chloroplast, and mitochondria OTUs were removed following annotation. OTUs were clustered with a 97% similarity cutoff using UPARSE (version 7.1, http://drive5.com/uparse/ 6 September 2024), and chimeric sequences were identified and removed using UCHIME. The taxonomy of each 16S rRNA gene sequence was analyzed by the RDP Classifier algorithm (http://rdp.cme.msu.edu/ 6 September 2024) against the Silva 16S rRNA database using a confidence threshold of 70%.

### 2.3. Biodiversity Analysis

Alpha diversity indices, including Chao and Ace indices (richness estimate) and Shannon index (diversity estimate) in the samples were analyzed from the sequencing data using Mothur software (version 1.30.2) to estimate the richness and diversity of microbial communities. The mean values of these indices were compared among groups using ANOVAs in R (version 3.3.1), followed by Tukey’s post hoc HSD test to obtain *p*-values for pairwise comparisons across each life stage. Rarefaction curves, the depth of sampling of a community compared with its total diversity, were constructed using R software (version 3.3.1). Beta diversity measurements, the differences in species composition between assemblages or regions, were visualized using R software (version 3.3.1) for the principal coordinate analysis (PCoA). The beta diversity distance matrix was calculated by QIIME (version 2020.2.0), and a hierarchical clustering analysis was performed according to the beta diversity distance matrix. The UPGMA (Unweighted Pair-group Method with Arithmetic Mean) was used to construct the tree structure, and then R was used to draw the tree to visualize the similarity or difference in community composition in different samples. The composition and abundance distribution tables of each sample at phylum and genus levels were obtained by QIIME software (version 1.9.1). For differences in microbial community composition across the six life stages, ANOSIMs in QIIME were performed. To determine which stages were most similar to one another, R values from each model were used. Here, all models were assessed with a permutation test (999 permutations), and FDR-corrected *p*-values were provided to correct for multiple comparisons. Linear discriminant analysis effect size (LEfSe) was performed to identify differentially abundant bacteria among different groups with a linear discriminant analysis (LDA) score threshold >4 [60]. The PICRUSt analysis was based on the Kyoto Encyclopedia of Genes and Genomes (KEGG) to query protein sequences and predict gene functions. The predicted gene functions showed different abundance metabolic pathways between groups. An ANOVA was performed to determine the significant differences in KEGG pathways across groups. In this study, the bioinformatic analysis of the gut microbiota was carried out using the Majorbio Cloud platform (https://cloud.majorbio.com 6 September 2024).

## 3. Results

### 3.1. Summary of Sequencing Data

**Figure 1:**
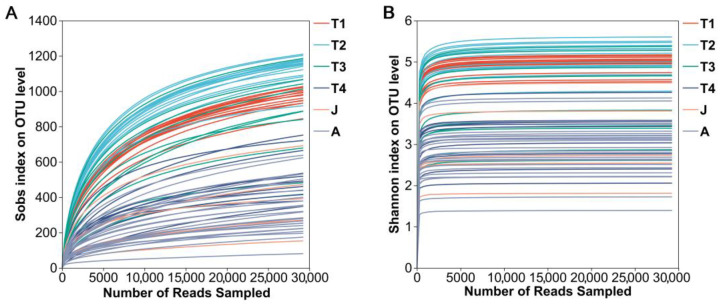
Rarefaction curves of samples. (**A**): Sobs index; (**B**): Shannon index. T1: Gosner 18–31; T2: Gosner 32–41; T3: Gosner 42–44; T4: Gosner 45–46; J: juvenile; A: adult.

A total of 4,068,104 high-quality reads were obtained from all gut microbiota samples at six developmental stages. They were grouped into 2063 OTUs (>97% sequence similarity), which encompassed 30 phyla affiliated with 312 genera. Both rarefaction curves (Sobs rarefaction curve and Shannon index rarefaction curve) suggested that the sequencing depth was sufficient to cover most bacterial communities in all samples (Figure 1A,B).

### 3.2. Gut Microbial Diversity

#### 3.2.1. Alpha Diversity of Gut Microbiota

**Figure 2:**
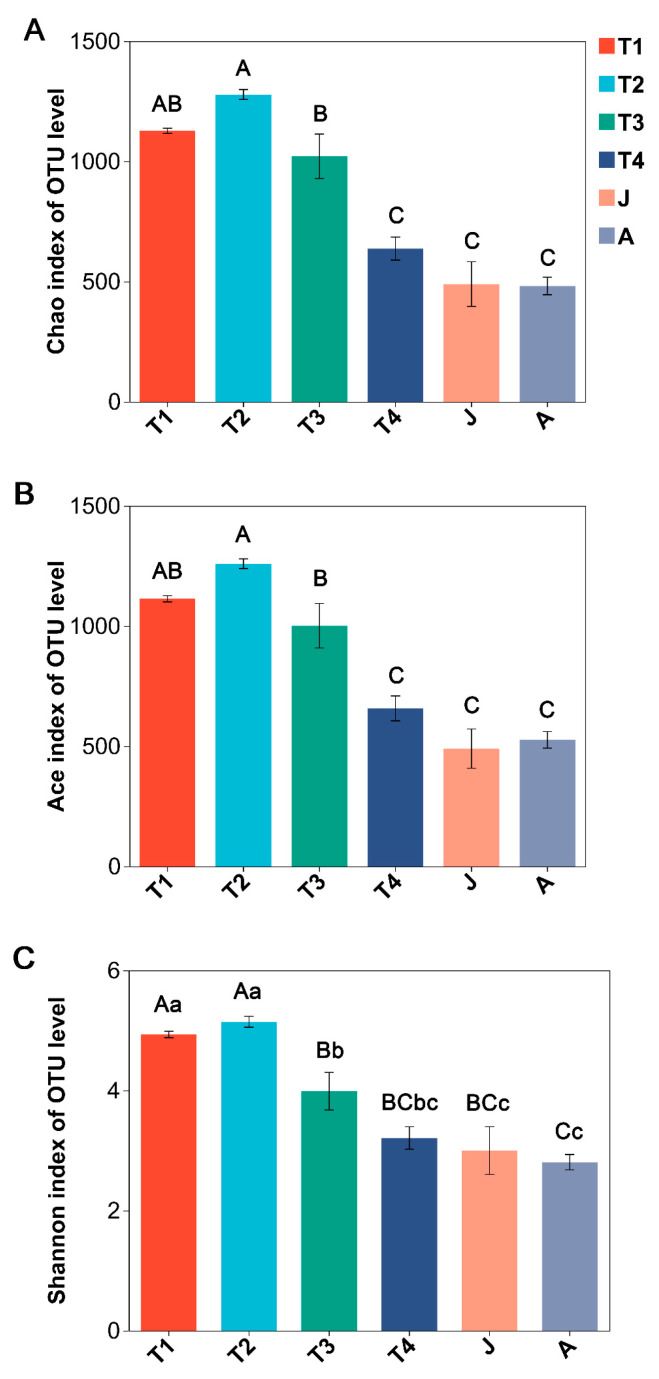
Comparison of alpha diversity of intestinal microbiota at different growth stages of *B. tibetanus.* (**A**): Chao index; (**B**): Ace index; (**C**): Shannon index. Different letters indicate significant differences among groups. Capital letters represent extremely significant differences (*p* < 0.01), and lowercase letters represent significant differences (*p* < 0.05).

Significant differences were observed among all developmental groups in terms of Chao, Ace, and Shannon indices values (ANOVA, all *p* < 0.001; Figure 2A–C). These three indices of the T2 group were the highest and decreased as development progressed, and all their values were the lowest in the A group (Figure 2A–C). The Chao, Ace, and Shannon indices were significantly decreased in the J group compared to the T2 group, whereas no significant differences existed between T4 and J and J and A groups (HSD’s test: all *p* > 0.05).

#### 3.2.2. Beta Diversity Analysis

**Figure 3:**
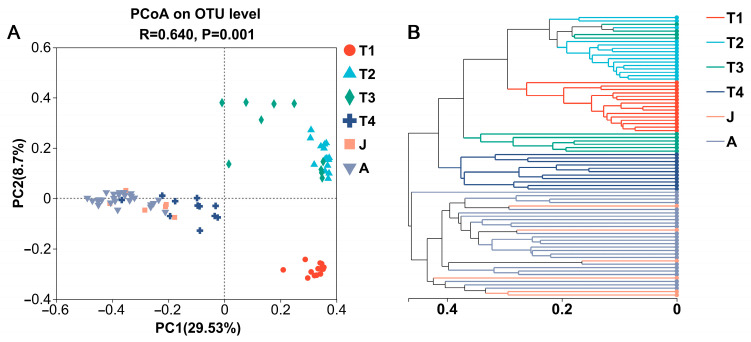
PCoA plot based on Bray–Curtis distances (**A**) and hierarchical clustering tree (**B**) of gut microbes at different growth stages. T1: Gosner 18–31; T2: Gosner 32–41; T3: Gosner 42–44; T4: Gosner 45–46; J: juvenile; A: adult.

At the OTU level, a PCoA based on Bray–Curtis dissimilarities demonstrated most samples clustered according to development stages and significant differences in bacterial community structure for different groups (ANOSIM, *R* = 0.640, *p* = 0.001; Figure 3A). The results showed clear separations among the six groups with different developmental stages, except for many overlaps in the T2 and T3 groups. Further, the gut microbiota of the J group was similar to those of the T4 and A groups. Similar results were produced by hierarchical clustering (Figure 3B), implying that the distribution of gut microbiota in the toad became more similar after metamorphosis.

### 3.3. Gut Microbial Composition

#### 3.3.1. Community Composition of Gut Microbiota at the Phylum Level

**Figure 4:**
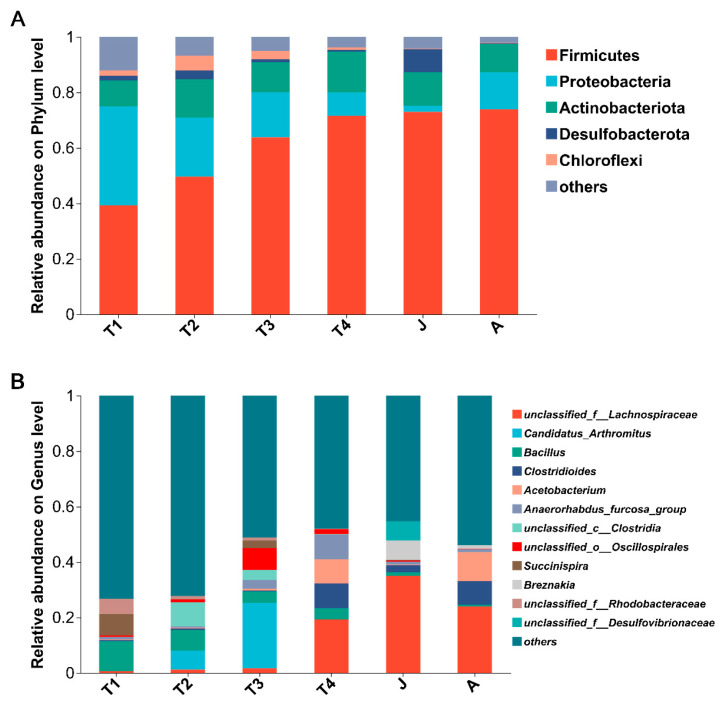
Community barplot analysis showing the relative abundance at the phylum (**A**) and genus (**B**) level at different growth stages. Abundances of phyla or genera less than 1% were classified as “others”. T1: Gosner 18–31; T2: Gosner 32–41; T3: Gosner 42–44; T4: Gosner 45–46; J: juvenile; A: adult.

There were 30 phyla detected from the gut microbiota of *B. tibetanus* in all developmental groups, with 7 phyla having a relative abundance of more than 1% (Figure 4A). For the T1 group, the gut microbiota was dominated by Firmicutes (39.2%), Proteobacteria (35.8%), Actinobacteria (9.3%), and Bacteroidetes (4.9%) (Figure 4A). Compared with the T1 group, the relative abundance of Bacteroidota in the T2 and T3 groups decreased by less than 0.2%. For the T2 group, the relative abundances of Firmicutes (from 39.2% to 49.6%), Actinobacteria (from 9.3% to 13.7%), and Cyanobacteria (from 2.0% to 2.7%) were increased compared to the T1 group, whereas that of Proteobacteria (from 35.8% to 21.4%) was decreased (Figure 4A). For the T3 group, the relative abundance of Firmicutes (from 49.6% to 63.8%) was increased compared to the T2 group, whereas those of Proteobacteria (from 21.4% to 16.3%), Actinobacteria (from 13.7% to 10.8%), and Cyanobacteria (from 2.7% to 1.6%) were decreased (Figure 4A). For the T4 group, the relative abundances of Firmicutes (from 63.8% to 71.7%) and Actinobacteria (from 10.8% to 14.8%) were increased compared to the T3 group, whereas those of Proteobacteria (from 16.3% to 8.3%) and Cyanobacteria (from 1.6% to 0.6%) were decreased (Figure 4A). For the J group, the relative abundances of Firmicutes (from 71.7% to 73.0%), Desulfobacterota (from 0.6% to 8.2%), and Bacteroidota (from 1.6% to 3.1%) were increased compared to the T4 group, whereas that of Actinobacteria (from 14.8% to 12.2%) was decreased (Figure 4A). For the A group, the relative abundance of Firmicutes (from 73.0% to 74.0%) was not significantly changed compared to that of the T4 group, whereas that of Proteobacteria (from 2.2% to 13.4%) and those of Actinobacteria (from 12.1% to 10.2%) and Bacteroidota (from 3.1% to 1.1%) were increased and decreased, respectively (Figure 4A).

**Figure 5:**
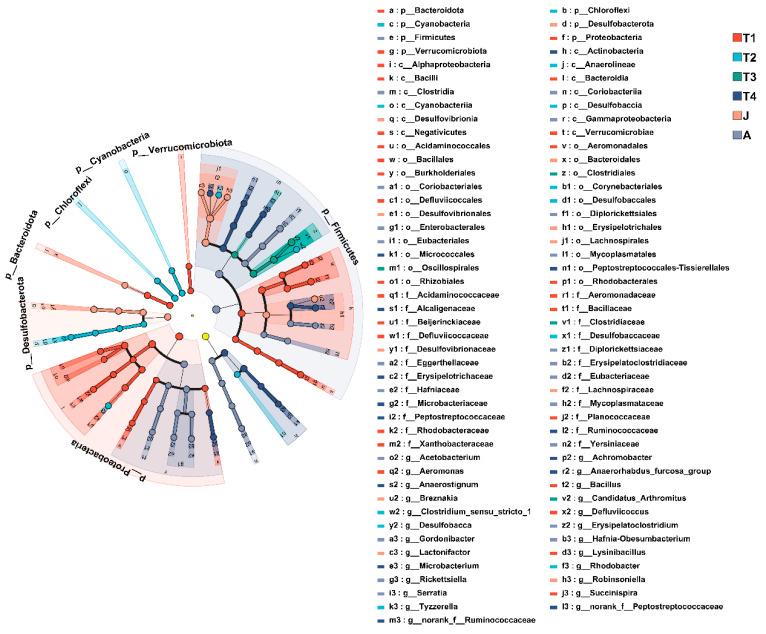
Cladogram of LEfSe analysis showing differentially abundant bacterial taxa in the gut microbiota among groups. The yellow node represents no significant difference between the sample groups. T1: Gosner 18–31; T2: Gosner 32–41; T3: Gosner 42–44; T4: Gosner 45–46; J: juvenile; A: adult.

**Figure 6:**
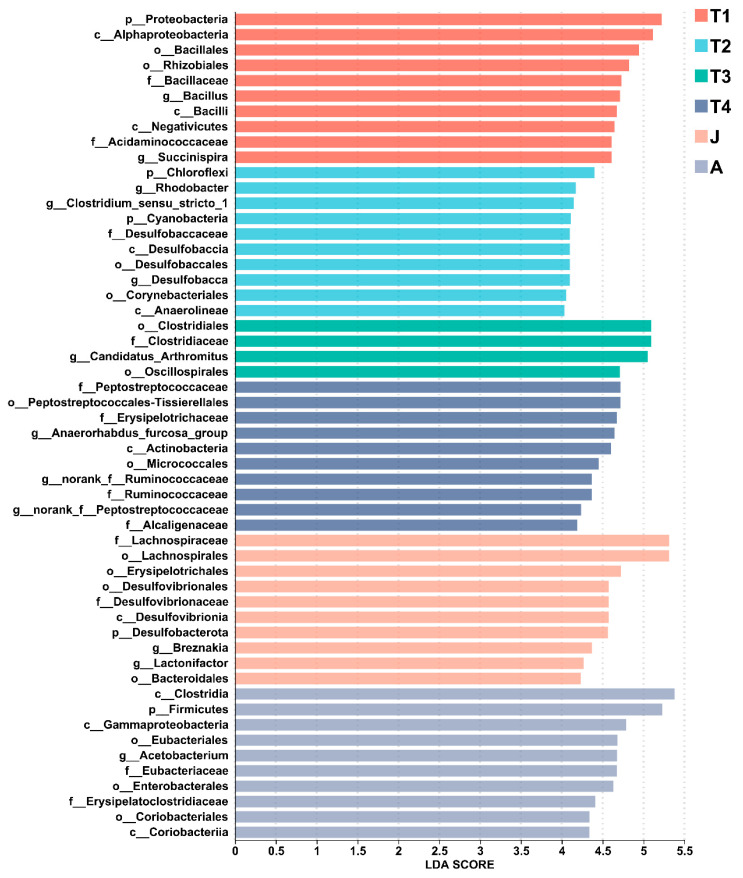
LEfSe analysis of the top 10 gut microbiota in all groups. LDA scores of differentially abundant taxa in the gut microbiota of the six groups from the LEfSe analysis. The top 10 dominant taxa in each group are shown. T1: Gosner 18–31; T2: Gosner 32–41; T3: Gosner 42–44; T4: Gosner 45–46; J: juvenile; A: adult.

At the phylum level, the composition of intestinal microbiota changed significantly with toad development. Twelve, or 40% of all the thirty phyla had high abundances (such as Firmicutes, Proteobacteria, Actinobacteria, Chloroflexi, Desulfobacterota, Bacteroidota, and Cyanobacteria) and had significant differences among the different stages of development (all *p* < 0.01 except Actinobacteria). Seven significantly differentiated phyla were identified by the LEfSe analysis, namely, Verrucomicrobiota (T1 group), Bacteroidota (T1 group), Proteobacteria (T1 group), Cyanobacteria (T2 group), Chloroflexi (T2 group), Desulfobacterota (J group), and Firmicutes (A group) (LDA > 4, *p* < 0.05; Figure 5 and Figure 6).

#### 3.3.2. Community Composition of Gut Microbiota at the Genus Level

There were 614 genera identified in all groups, with 20 genera having a relative abundance of more than 1% (Figure 4B). In the T1 group, the relative abundances of *Bacillus* (11.0%) and *Succinispira* (7.8%) were the highest. In the T2 group, the relative abundances of *g__unclassified_c__Clostridia* (from 0.03% to 8.7%) and *Candidatus_Arthromitus* (from 0.0% to 6.9%) were increased compared with those of the T1 group, whereas that of *Bacillus* (from 11.0% to 7.5%) was sharply decreased. In the T3 group, the relative abundances of *Candidatus_Arthromitus* (from 6.9% to 23.7%) and *g__unclassified_o__Oscillospirales* (from 1.0% to 7.8%) were increased compared with those of the T2 group, whereas that of *Bacillus* (from 7.5% to 4.1%) was decreased. In the T4 group, the relative abundances of *g__unclassified_f__Lachnospiraceae* (from 1.6% to 19.3%) and *g__Anaerorhabdus_furcosa_group* (from 3.3% to 9.0%) were increased compared with those of the T3 group, whereas those of *Candidatus_Arthromitus* (from 23.7% to 0.02%) and *g__unclassified_o__Oscillospirales* (from 7.8% to 1.7%) were decreased. In the J group, the relative abundances of *g__unclassified_f__Lachnospiraceae* (from 19.3% to 35.0%) and *g__unclassified_o__Micrococcales* (from 2.5% to 3.8%) were increased compared with those of the T4 group, whereas those of *g__unclassified_o__Oscillospirales* (from 1.7% to 0.4%) and *g__Anaerorhabdus_furcosa_group* (from 9.0% to 1.1%) were decreased. In the A group, the relative abundances of *Acetobacterium* (from 0.2% to 10.5%) and *g__unclassified_f__Lachnospiraceae* (from 35.0% to 24.1%) were increased and decreased compared with those of the J group, respectively.

At the genus level, LEfSe found significant enrichment of Succinispira, Aeromonas, and Defluviicoccus in the T1 group; Tyzzerella, Clostridium_sensu_stricto_1, Rhodobacter, and Desulfobacca in the T2 group; Candidatus_Arthromitus in the T3 group; Anaerostignum, norank_f_Peptostreptococcaceae, norank_f_Ruminococcaceae, Anaerhabdus_furcosa_group, Microbacterium, and Achromobacter in the T4 group; Lactonifacter, Robinsoniella, and Breznakia in the J group; and Acetobacterium, Erysipelatoclostridium, and Gordonibacter in the A group (LDA > 4, *p* < 0.05; Figure 5 and Figure 6).

### 3.4. Microbiota Functional Prediction Analysis

**Figure 7:**
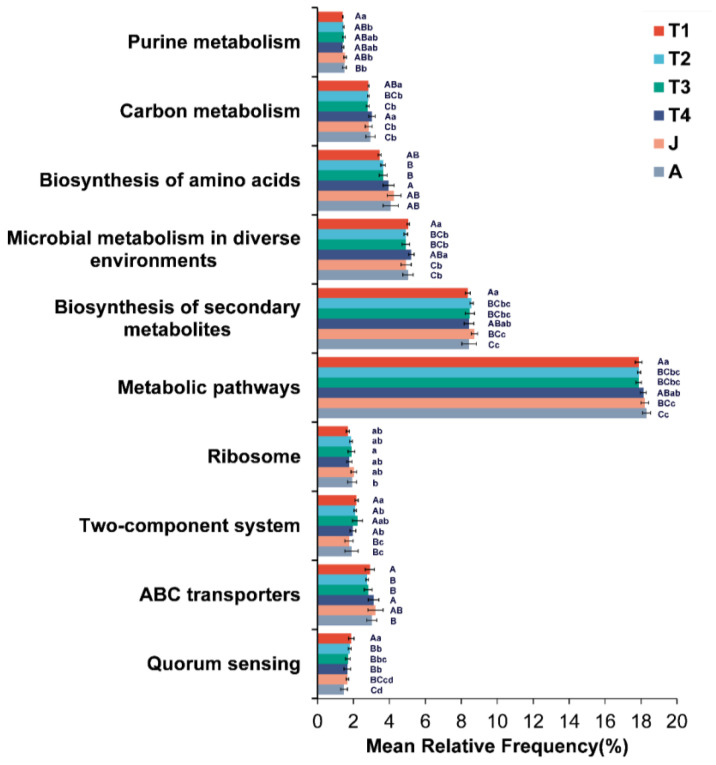
Predicted functions of the intestinal microbiota on KEGG pathways. T1: Gosner 18–31; T2: Gosner 32–41; T3: Gosner 42–44; T4: Gosner 45–46; J: juvenile; A: adult. Different letters indicate significant differences among groups. Capital letters represent extremely significant differences (*p* < 0.01), and lowercase letters represent significant differences (*p* < 0.05).

KEGG pathways were used to predict the functions of the intestinal microbiota at different developmental stages. The enriched KEGG pathways illustrated that the metabolic pathways and the biosynthesis of secondary metabolites were significant enrichment in all stages (Figure 7). For KEGG pathway level 3, pathways associated with Microbial metabolism in diverse environments, Quorum sensing, Two-component system and Purine metabolism were significantly enriched in the T1 group, the pathway associated with Ribosome was more enriched in the T3 group, and pathways associated with ABC transporters and Carbon metabolism were more enriched in the T4 group.

## 4. Discussion

There are few systematic studies on the ecological succession of intestinal microbiota during the development of anurans. Here, we provided a baseline description of the developmental succession of gut microbiota in the Tibetan toad by using a 16S rRNA sequencing analysis. The three alpha diversity indices were highest in the T2 group (Gosner 32 to 41), decreased as development progressed, and became stable at juvenile and adult stages. The developmental stage and feeding habitat could underlie this trend. Further, we found that the gut microbiota structure differed among different development stages of *B. tibetanus* and became relatively stable after metamorphosis. This developmental adaptation can be important for hosts grown with a change in habit use pattern.

Animal growth and development are tightly correlated with changes in intestinal microbiota [26,48,61]. Throughout the toad’s development, the intestinal microbiota of *B. tibetanus* undergoes changes. A higher alpha diversity was found in the T1 and T2 groups (Gosner 18 to 41), and its value decreased with the development of the toad, which was consistent with the results of *Andrias davidianus* in Brazil and Madagascar anuran faunas [40,62]. The intestinal microbiota of tadpoles is susceptible to external factors (e.g., growth, diet, temperature, drug, etc.), which cause it to be unstable, thus forming the characteristics of a high diversity [2,35,50,63,64,65]. In addition, the beta diversity analysis of the intestinal microbiota of *B. tibetanus* showed differences in different development stages of the toad. The intestinal microbiota of the T1 group was obviously separated from that of the other groups. However, the structure of the intestinal microbiota in the T2 and T3 groups tended to be similar, as well as that of the T4, juvenile, and adult groups. These results are similar to the research on *Rana chensinensis*, *Bufo gargarizans*, *Andrias davidianus*, and zebrafish gut microbiota with high-throughput sequencing [62,66,67,68]. Tadpoles during late developmental stages (T3 and T4 group, after Gosner stage 41) were in the metamorphosis period, and their functional traits exhibited significant changes to adapt switching from water to land [66,67]. In this process, the feeding of tadpoles was decreased, which had an impact on the colonization of intestinal microbiota [66,69,70,71,72]. Many studies have demonstrated that diet has a substantial impact on gut microbiota [73,74,75,76]. These changes in gut microbiota could be caused by the direct effect of diet composition [77]. In many animal taxa, intestinal microbiota undergo dynamic changes due to physical, morphological, and chemical changes concomitantly with the development of the digestive system [32,78]. The complex life history of most amphibians consists of an aquatic larval stage followed by terrestrial juvenile and adult stages [79]. In addition to diet, the structure and function of intestinal microbiota are more likely to be influenced by changes in the living environment [80,81].

The abundance of intestinal microbiota in different growth stages of *B. tibetanus* constantly changed. The dominant phyla of *B. tibetanus* tadpoles were Firmicutes, Proteobacteria, Actinobacteriota, and Desulfobacterota, which were inconsistent with those of *B. gargarizans*, *Rana pipiens* and *Rana dybowskii* tadpoles [48,63,67]. The inconsistent results may be attributed to the diversity of the dietary habits of tadpoles [69,82,83]. *B. tibetanus* lives in meadow wetlands on the Qinghai-Xizang Plateau, where there are few phytoplankton and more benthic animal species [54,84,85,86]. Proteobacteria was the most abundant phylum in the gut of *B. tibetanus*, and it was enriched in the gut microbiota of the T1 group and gradually decreased with growth. Our results are consistent with those of Mirpuri et al., (2014) [87], who found that there was a transient dominance of Proteobacteria in newborn mice, and it was progressively lost with age. Similarly, a study on the intestinal microbiota of *L. pipiens* tadpoles showed that the proportion of Proteobacteria was high in the tadpole period [32]. Proteobacteria can participate in the decomposition of complex carbohydrates in animal intestines and convert them into small molecules that can be absorbed and utilized by animals [88,89,90]. At this stage, tadpoles grow rapidly, thus the enrichment of Proteobacteria is beneficial for them to access external nutrients.

Desulfobacterota was the most abundant phylum in the gut of *B. tibetanus* and was enriched in the gut microbiota of the J group by the LEfSe analysis. Desulfobacterota has the ability to reduce sulfate to hydrogen sulfide, which can help them to deal with some sulfur-containing compounds. The generated hydrogen sulfide can regulate the redox state in the intestine to a certain extent, and may participate in signal transmission in some physiological processes [91,92]. This may suggest this toad consumes more sulfur-containing foods in the juvenile stage, and being rich in Desulfobacterota can increase its ability to digest and help it adapt to the plateau environment [93,94]. In addition, Desulfobacterota is associated with a hypoxic environment [95]; therefore, it is more likely to evolve this characteristic to survive in the intestine.

Adult toads, compared to tadpoles, had a higher relative abundance of Firmicutes and a reduced abundance of Bacteroidetes. Firmicutes and Bacteroidetes are the predominant bacterial phyla colonizing the healthy intestinal of humans and animals. Studies have shown that Firmicutes accounted for a high proportion of the gut microbiota in many animals [7,96,97,98,99,100,101]. For example, research on Chinese giant salamanders has found that the abundance of Firmicutes increases at the ages of 3 and 4 [102]. Similar to our results, Firmicutes was most abundant in *Polypedates megacephalus* and *L. pipiens* [32,103]. Many studies have shown that dietary fiber is one of the key factors influencing gut Firmicutes [104,105,106]. It has been found that Firmicutes are important in maintaining gut homeostasis, as they possess many genes responsible for fermenting dietary fiber and could interact with the intestinal mucosa [107]. Some studies have demonstrated that gut Firmicutes can produce byproducts through fermentation, such as short-chain fatty acids, that are directly absorbed through the host bowel wall [35,108,109,110]. This process may promote their metabolism and increase energy intake. Firmicutes may play a crucial role in the growth and development of *B. tibetanus.* Therefore, the significant changes in the abundance of Firmicutes between adults and tadpoles were likely to be caused by diet change. However, the most enriched phyla in the intestines of *Odorrana tormota*, *Odorrana graminea*, and *Amolops wuyiensis* were inconsistent with ours [111]. The composition of gut microbiota in different species is affected by factors such as host genotype, individual development stage, gender, habitat, captivity, and seasonal differences [111,112], which may lead to inconsistent results. At present, the factors affecting the intestinal flora of amphibians are mainly biased towards the environment [40]. The results of this study suggest that the dynamics of intestinal microbiota in Tibetan toads may be potentially related to the physiological characteristics of the host, but the deeper mechanisms underlying the changes in gut microbiota still need further verification.

## 5. Conclusions

In conclusion, the developmental stages play a vital role in reshaping the structure and function of the intestinal microbiota of *B. tibetanus.* The reshaped intestinal microbiota could be due to the growth and diet of this toad. Future research will need to better determine the role and molecular mechanisms of the growth stages behind these effects. Understanding these changes can provide new ideas for the protection of anurans.

## Data Availability

The raw sequence data information is available in the NCBI sequence reads archive with number PRJNA955012.
